# Factors controlling the mechanical properties degradation and permeability of coal subjected to liquid nitrogen freeze-thaw

**DOI:** 10.1038/s41598-017-04019-7

**Published:** 2017-06-16

**Authors:** Lei Qin, Cheng Zhai, Shimin Liu, Jizhao Xu

**Affiliations:** 1 Key Laboratory of Coal Methane and Fire Control, Ministry of Education, China University of Mining and Technology, Xuzhou, Jiangsu 221116 China; 2 School of Safety Engineering, China University of Mining and Technology, Xuzhou, Jiangsu 221116 China; 3 Department of Energy and Mineral Engineering, G3 Center and Energy Institute, Pennsylvania State University, University Park, Pennsylvania 16802 United States

## Abstract

Freeze-thaw induced fracturing coal by liquid nitrogen (LN_2_) injection exerts a significant positive effect on the fracture permeability enhancement of the coal reservoir. To evaluate the different freeze-thaw variables which modify the mechanical properties of treated coals, the effects of freezing time, number of freeze-thaw cycles, and the moisture content of coal were studied using combined uniaxial compression and acoustic emission testing systems. Freezing the samples with LN_2_ for increasing amounts of time degraded the strength of coal within a certain limit. Comparison to freezing time, freeze-thaw cycling caused much more damage to the coal strength. The third variable studied, freeze-thaw damage resulting from high moisture content, was restricted by the coal’s moisture saturation limit. Based on the experimental results, equations describing the amount of damage caused by each of the different freeze-thaw variables were empirically regressed. Additionally, by using the ultrasonic wave detection method and fractal dimension analyses, how freeze-thaw induced fractures in the coal was quantitatively analyzed. The results also showed that the velocity of ultrasonic waves had a negative correlation with coal permeability, and the freeze-thaw cycles significantly augment the permeability of frozen-thawed coal masses.

## Introduction

For fracturing coal using an anhydrous fluid, liquid nitrogen (LN_2_) is attracting increasing attention^1–3^. The feasibility of this fracturing technology has been studied for the exploitation of unconventional oil and gas^4–6^. An unconventional gas, the worldwide reserves of coal-bed methane (CBM) are very large^7, 8^. High-efficiency extraction and utilization of CBM not only can decrease greenhouse gas emission and avoid gas disasters in coal mines but can also augment the world’s energy supply^9–12^.

Because deep coal seams have low permeability and adsorb gases, the coal formations have to be fractured and gas seepage pathways opened before extracting CBM^13–15^. Compared with hydraulic fracturing and gas fracturing^16, 17^, fracturing using LN_2_ shows advantages by both modifying the coal seam and increasing CBM production. Under ambient pressures, the temperature of LN_2_ can be as low as −196 °C and it produces a latent heat of vaporization of 5.56 kJ/mol. In addition, LN_2_ expands 696 times upon vaporization, resulting in the generation of strong expansion forces^18^. Moreover, as coal seams are commonly saturated with groundwater, moisture in coal cleats freezes quickly after cooling where coal is in contact with LN_2_. The water-ice phase transition causes a volume expansion of 9% and in theory this produces up to 207 MPa of frost heaving force on a crack’s tip^19^.

With respect to fracturing using LN_2_, in the 1990’s McDaniel *et al*.^6^ and Grundmann *et al*.^5^ used LN_2_ as a fracturing medium to improve the production of oil and gas. Based on previous studies, Li *et al*. designed a fracturing method using the vaporization of LN_2_ applicable to increasing the production of shale gas^3^. Applying LN_2_ as a cooling medium, Coetzee *et al*.^4^ and Cai *et al*.^20^ found that low-temperature freezing by LN_2_ can efficiently promote the development and connection of pores and fractures.

Currently, most research using LN_2_ on coal is focused on the feasibility of freeze-thaw fracturing using LN_2_. There has been no systematic study on how different freeze-thaw variables modify the mechanical properties of the coal. Based on previous studies, the authors put forward a fracturing technology of freeze-thaw cycling using LN_2_ combined with fracturing technologies for extracting CBM^18^. Under the triple effects of LN_2_’s low-temperature, the vaporization and expansion of LN_2_, and the volume expansion due to the water-ice phase transition, strong freeze-thaw cycles occurred in the coal and these cycles both decreased the coal’s mechanical strength and produced a fracture network. This provided very favorable reservoir stimulations for extracting CBM.

This paper focuses on studying the control factors influencing the changes in mechanical properties and fracture evolution of frozen-thawed coal samples with LN_2_. The variables investigated are LN_2_ freezing time, the number of freeze-thaw cycles, and the moisture content of the coal. This research attempts to explore the relationships between mechanics parameters of low-rank coal and freeze-thaw variables. The goal is to provide parametric study for freeze-thaw fracturing technology that can be implemented in CBM reservoirs in near future.

## Results

### Stress-strain curves

**Figure 1:**
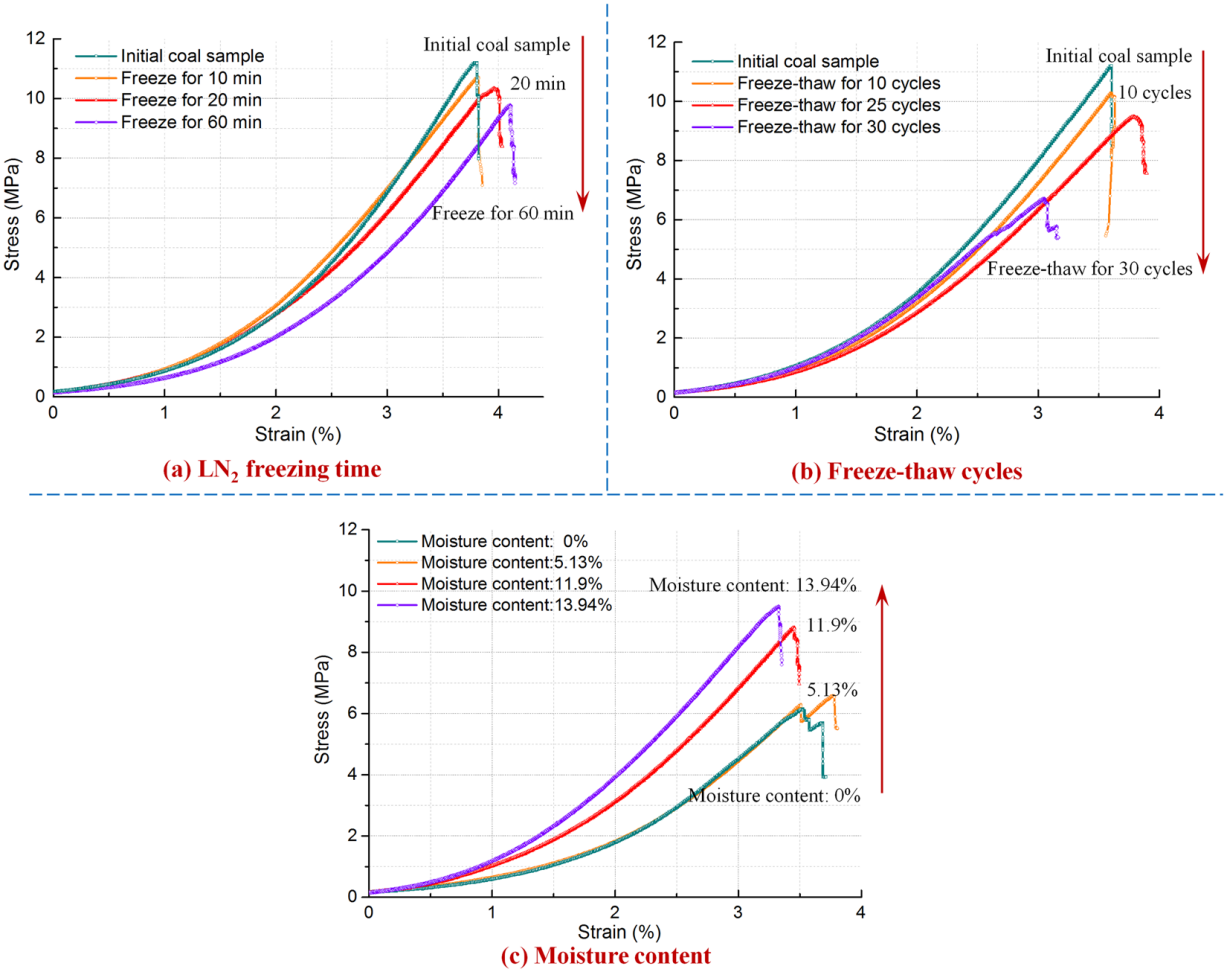
Uniaxial stress-strain curves for coal samples subjected to three different freeze-thaw variables. The stress-strain curves for the three variables shown in: (**a**) LN_2_ freezing time; (**b**) freeze-thaw cycles; (**c**) coal’ moisture content.

Uniaxial compression tests were performed to evaluate the strength of tested coals and to determine how the coal deformed and failed under the designated experimental conditions. The strength and deformation results were obtained by measuring the physical properties of frozen-thawed coal with different moisture contents and after coal samples had been subjected to the different freezing time durations and different numbers of freeze-thaw cycles as described in the experimental method section. Moisture in coal cleats froze and expanded and numerous open-type freeze-thaw induced fractures were formed by frost heaving. These fractures were compressed and closed again during uniaxial compression loading but new compression fractures were generated and caused additional structural damage. The coal samples that underwent different freeze-thaw procedures suffered different degrees of structural damages. The uniaxial compression experiments plus the acoustic emissions allowed the development of the induced fractures to be monitored^21^. In this way, the main factors influencing the freeze-thaw damage in the coal and how these factors changed the mechanical properties of the coal could be assessed (Fig. 1).

Figure 1 shows the uniaxial stress-strain curves for the coal samples subjected to different freeze-thaw variables. The uniaxial compressive strength of the original non-frozen coal samples was 11.2 MPa. After the samples were frozen by LN_2_ for 20 and 60 min, the compressive strengths were 10.3 and 9.7 MPa, respectively, declining by 8% and 13.4% (Fig. 1(a)). When the cyclic LN_2_ freeze-thaw treatment was administered for 10 and 30 cycles, the compressive strengths were 10.28 and 6.7 MPa, decreasing by 8.2% and 40.2%, (Fig. 1(b)). For the coal samples with moisture contents of 13.9%, 11.9% and 0%, after being frozen once by LN_2_ for 90 min their compressive strengths were 9.4, 8.8, and 6.1 MPa, respectively, strengths that dropped by 16.1%, 21.4% and 45.5% as shown in Fig. 1(c). Therefore, uniaxial compressive strengths of the frozen-thawed coal presented a negative correlation with LN_2_ freezing time and freeze-thaw cycles while a positive correlation with moisture contents in coal.

### Axial and hoop strains

**Figure 2:**
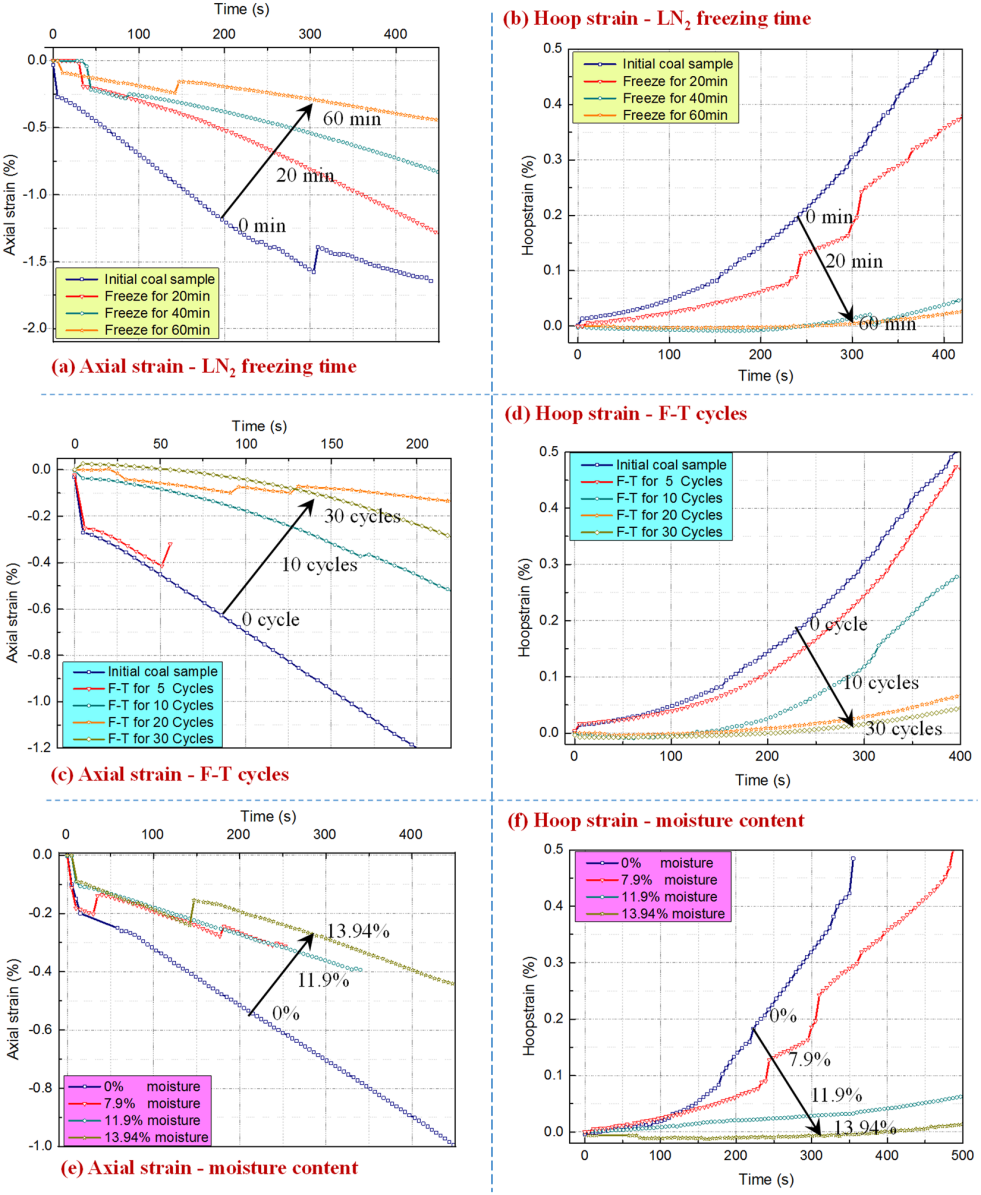
Influence of freeze-thaw variables on strains of the coal masses in the compression process. (**a**), (**c**), and (**e**); axial strains; (**b**), (**d**), and (**f**); hoop strains.

The axial and hoop strains of the frozen-thawed coal samples were measured during uniaxial compression (during compression, axial strains are negative, hoop strains are positive). The relationships between axial strain and the freeze-thaw variables are shown in Figs. 2(a), (c), and (e) and those between hoop strain and the variables are illustrated in Figs. 2(b), (d), and (f). For the coal sample tests, the axial loading suddenly applied to coal and this initiates a shock stress on the sample which induces the strain fluctuation at the early stage as shown in Figs. 2(a), (c), and (e). After the axial stress loaded evenly, the strain evolution are smooth. When the uniaxial loading had run for 200 s, the axial strains were −1.2% for the raw coal, −0.5% and −0.19% for the coal frozen once for 20 and 60 min, −0.45% and −0.23% for the coal frozen-thawed for 10 and 20 cycles, and −0.52% and −0.2% for the coal with moisture contents of 0% and 13.9% frozen for 90 min. At the 200 s mark, the hoop strains for the raw coal was 0.15% and the hoop strains were 0.06% and 0.002% for the 20 and 60 min frozen coal, 0.12% and 0.03% for the 10 and 20 cycle frozen coal, and 0.14% and −0.01% for the 0% and 13.9% moisture coal. The absolute values of axial strains and hoop strains decreased with increasing freezing time, number of freeze-thaw cycles, and moisture content (Fig. 2). This means that the frozen-thawed coal more easily damage with the increase of the three variables. This is because the frost heaving effect of the moisture in the cleats becomes stronger as these three factors increase and this weakens the coal. Among these three factors, the number of freeze-thaw cycles damages the coal at most.

### Acoustic emission measurements

Uniaxial compression causes stress concentrations and slippage friction around freeze-thaw induced fractures. Some of the strain energy in the coal is released in the form of elastic waves and these waves can be detected as acoustic emissions (AE)^22^. By collecting the acoustic emission signals from the frozen-thawed coal during the compression, the extension, connection, and failure of the freeze-thaw induced fractures in the coal can be inferred^23–25^.

A few micro-fractures existed in the original coal samples. Additional fractures at many scales were generated in the coal samples by the LN_2_ freeze-thaw treatments. Then the freeze-thaw induced fractures were compacted during uniaxial compression and the compression generates even more fractures when the original freeze-thaw induced fractures reached their ultimate strengths. Subsequently, fractures connected and degrade the coal samples to even more extent. The frozen-thawed coal produced a large number of acoustic emissions at different stages of compression including friction and compression signals, cracking signals, and splitting signals.

**Figure 3:**
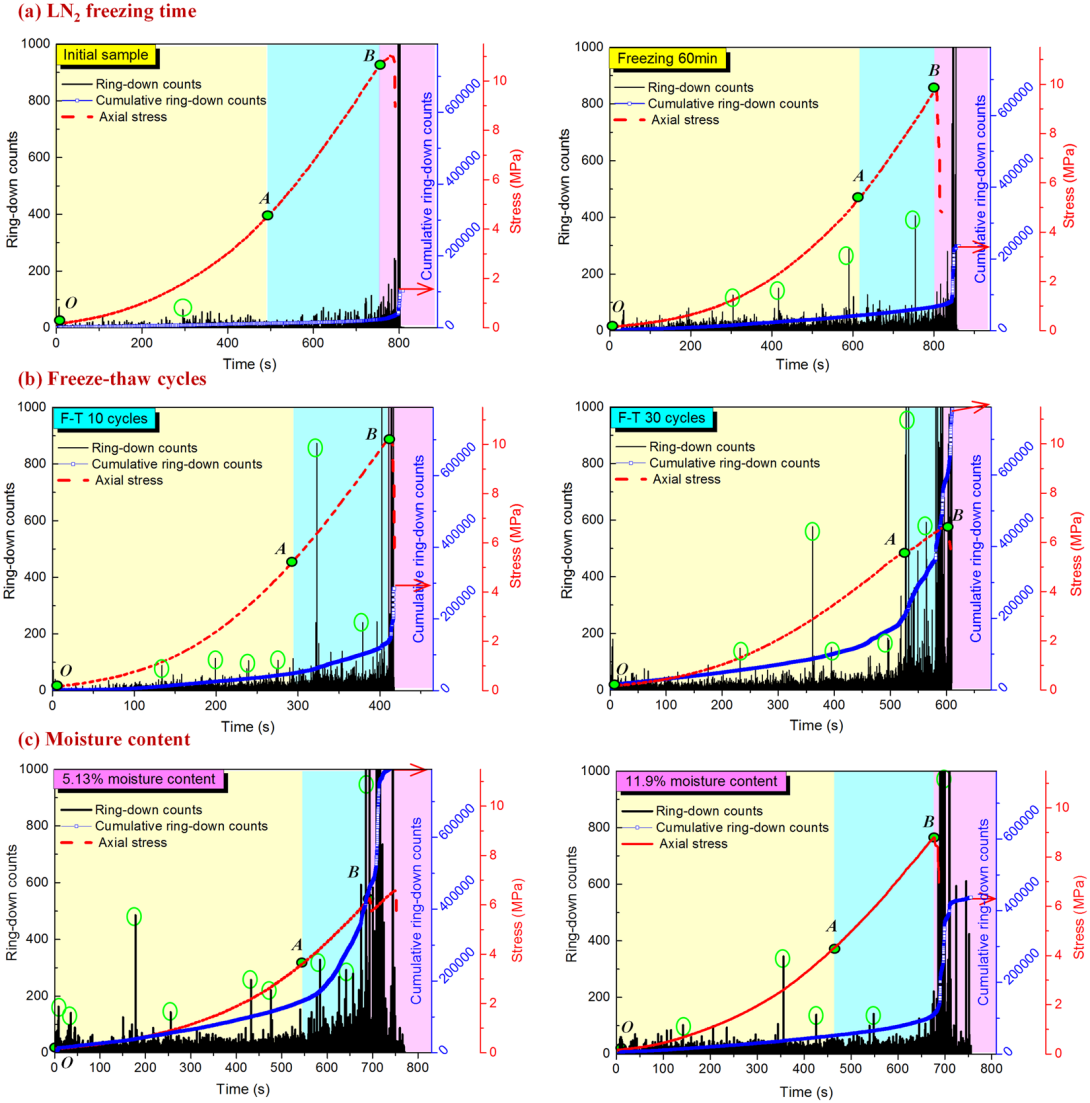
Acoustic emissions and uniaxial compression strengths for frozen-thawed coal. (**a**) LN_2_ freezing time; (**b**) freeze-thaw cycles; (**c**) coal’ moisture content.

In this study, the compression failure of the frozen-thawed coal was divided into three stages: compaction, elastic deformation and yield failure stages. Figure 3 shows the compaction stages and lines OA and AB represent the fracture compaction and elastic deformation stages, respectively. The stage after point B represents the yield and failure stage of the coal. According to the acoustic emission and uniaxial compression curves in Figs. 3(a) and (b), with longer freezing times and a greater number of freeze-thaw cycles, the crack compaction stage was prolonged whereas the elastic stage was shortened and the compressive peak strengths declined. In addition, a sudden increase of acoustic emissions (marked by green circles in the figure) and total ringing counts of the samples increased with longer freezing times and the number of freeze-thaw cycles. Conversely, as the moisture contents in the coal increased, the crack compaction stage shortened but the elastic stage lengthened and the compressive strengths increased. In addition, the sudden increase of acoustic emission signals (marked by green circles in the figure) and total ringing counts of samples dropped, as shown in Fig. 3(c). These acoustic emission results showed that with increasing freezing time and number of freeze-thaw cycles, the freeze-thaw induced damage gradually increased. In addition, the number of the acoustic emission signals from the coal subjected to freeze-thaw cycles was more than that had been subjected to only a single long-time freezing treatment. Higher moisture contents in the frozen-thawed coal promoted its mechanical strength because the moisture supported the fractures. However, the frost heaving effect in the coal was heightened by the increased moisture so the higher water content caused more freeze-thaw damage. In general, completely saturated coal contains 10–15% water, so coal freeze-thaw damage related to moisture contents is limited by its fully saturated water content.

## Discussion

### Changes in frozen-thawed coal mechanical properties

**Figure 4:**
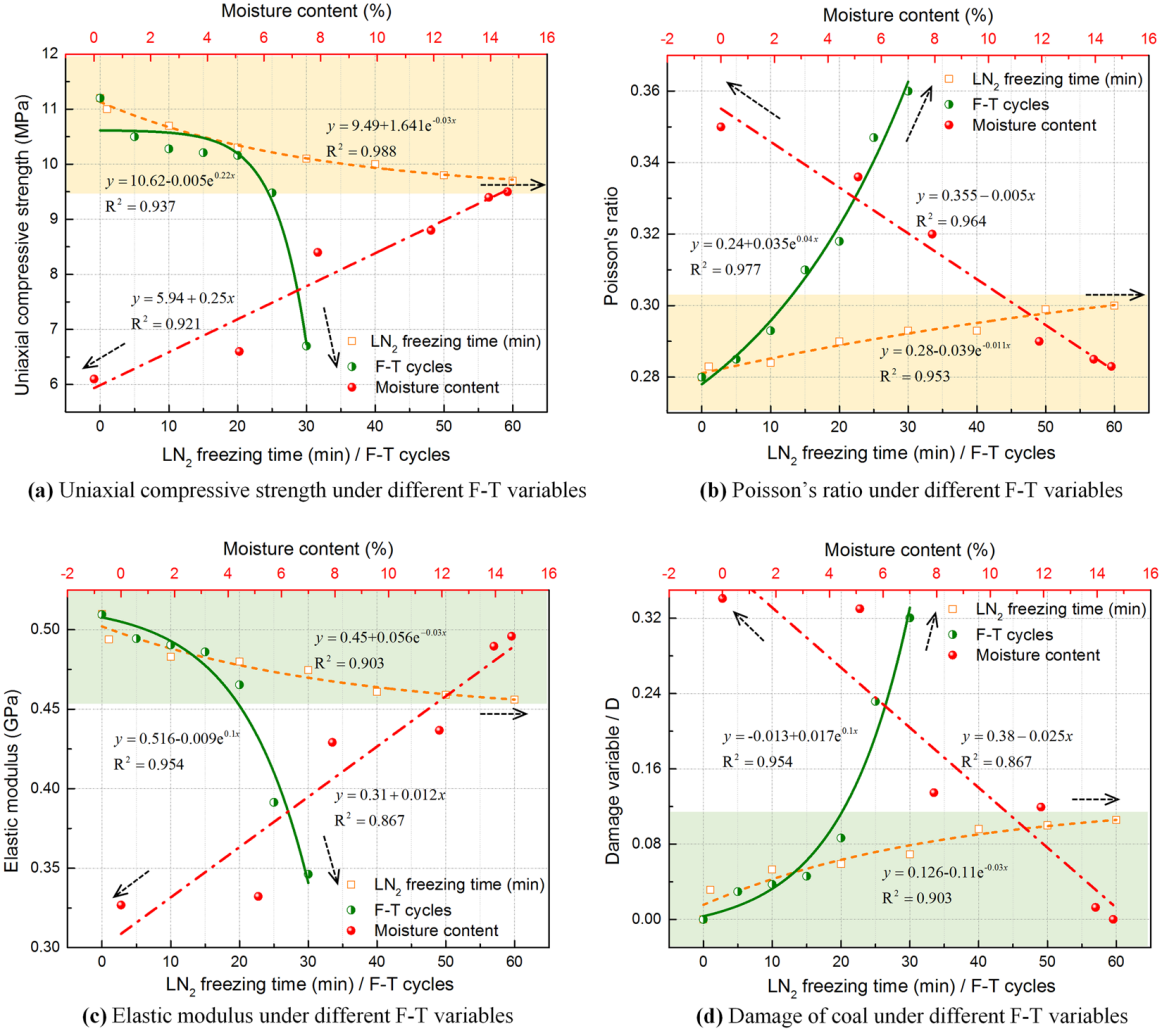
Graph showing mechanics parameters of frozen-thawed coal samples subjected to different freeze-thaw variables. (**a**) uniaxial compressive strength; (**b**)) Poisson’s ratio; (**c**) elastic modulus; (**d**) damage variable.

Uniaxial compressive strength refers to the ultimate stress on a sample at failure under uniaxial compression. The smaller the uniaxial compressive strength, the easier the coal can be damaged under the load. The relationship between uniaxial compressive strength of the frozen-thawed coal and freeze-thaw variables (LN_2_ freezing time, number of freeze-thaw cycles and moisture content in the coal) is shown in Fig. 4(a). Compared with a virgin sample of coal, the compressive strengths of the coal samples frozen once for 60 min and the samples subjected to 30 cycles of freeze-thawing decreased by 13.4% and 40.2%, respectively. Obviously, the freeze-thaw cycles caused more damage to the coal than did a single freezing event. Compared with a non-frozen coal sample, the compressive strengths of the coal samples with the moisture contents of 0% and 13.9% declined by 45.5% and 15.2%, respectively, after those samples were frozen once for 90 min (Fig. 4(a)). Therefore, the uniaxial compressive strengths of the frozen-thawed coal masses exhibited a negative correlation with LN_2_ freezing time and freeze-thaw cycles while a positive correlation with moisture contents in coal. Equations fit to the relationship between the three freeze-thaw variables and the ultimate uniaxial compressive strength for these coal samples are listed in Table 1.

**Table 1:** Equations fit to the relationships between mechanics parameters of frozen-thawed coal and freeze-thaw variables.

Mechanics parameters	Freeze - thaw variables	Fitting Formula	Model	Adj. R-Square
Uniaxial compressive strength (MPa)	*T*	*y* = 9.49 + 1.641e^−0.03*T*^	Exponential	0.988
	*C*	*y* = 10.62 − 0.005e^0.22*C*^	Exponential	0.937
	*w*	*y* = 5.94 + 0.25*w*	Line	0.921
Poisson’s ratio	*T*	*y* = 0.28 − 0.039e^−0.011*T*^	Exponential	0.953
	*C*	*y* = 0.24 + 0.035e^0.04*C*^	Exponential	0.977
	*w*	*y* = 0.355 − 0.005*w*	Line	0.964
Elastic modulus (GPa)	*T*	*y* = 0.45 + 0.056e^−0.03*T*^	Exponential	0.903
	*C*	*y* = 0.516 − 0.009e^0.1*C*^	Exponential	0.954
	*w*	*y* = 0.31 + 0.012*w*	Line	0.867

*Note*: *T*-LN_2_ freezing time (min); *C*-Freeze-thaw cycles; *w*- Moisture content of coal samples (%).

Poisson’s ratio is the ratio between absolute transverse strain and absolute axial strain when a test specimen is subjected to uniaxial force. Poisson’s ratio reflects the elastic constant for materials in transverse deformation. The larger the Poisson’s ratio, the easier a material expands or deforms when subjected to a load. The Poisson’s ratios of the frozen-thawed coal was positively correlated with freezing time and the number of freeze-thaw cycles but negatively correlated with moisture content. Compared with a non-frozen coal sample, the Poisson’s ratio of the samples frozen for one 60 min period and the samples frozen-thawed for 30 cycles increased by 7.14% and 28.6%, respectively. Poisson’s ratios for the coal with moisture contents of 0% and 13.9% increased by 25% and 1.8% after the coal was frozen once for 90 min, as shown in Fig. 4(b). LN_2_ Freezing time exerted a less effect on Poisson’s ratio while freeze-thaw cycles caused a larger and constantly aggravated degradation on Poisson’s ratio. The moisture content of the frozen-thawed coal was very important to the magnitude of the change in Poisson’s ratio. However, the absolute change in the ratio was controlled by the moisture saturation limit of the coal. Equations fit to the relationships between the three freeze-thaw variables and Poisson’s ratio are listed in Table 1.

The elastic modulus represents the capacity of a material to resist elastic deformation. The larger the elastic modulus is, the larger the stress that must be applied to a material for it to undergo a given amount of elastic deformation. In simple terms, the larger the elastic modulus is, the stiffer the material is. The elastic modulus of the frozen-thawed coal had a negative correlation with LN_2_ freezing time and the number of freeze-thaw cycles but a positive correlation with moisture content. Compared with a sample of non-frozen coal, the elastic moduli of the coal frozen once for 60 min and coal frozen-thawed for 30 cycles fell by 10.6% and 31.4%, respectively. The elastic moduli for the coal with moisture contents of 0% and 13.9% declined by 45.5% and 15.2% after the coal was frozen once for 90 min, as shown in Fig. 4(c). Equations fit to the relationships between the three freeze-thaw variables and the elastic modulus are listed in Table 1.

Numerous tensile and shear-type fractures were formed in the coal samples after being frozen because freezing the water in the cleats caused frost heaving and also because the low temperature caused minerals in the coal to shrink non-uniformly. It has been demonstrated in previous sections of this paper that different freeze-thaw variables caused different degrees of damage to the coal. According to the damage mechanics theory of Nemat-Nasser and Taya’s^26^, a freeze-thaw damage variable *D* can be defined as follows based on the elastic modulus:

1 $$D=1-\frac{{E}_{n}}{{E}_{{0}}}$$

where, *E*_*0*_ and *E*_*n*_ represent the elastic moduli of the original sample and the frozen-thawed coal sample.

Figure 4(d) shows the fitting curves for three freeze-thaw variables (LN_2_ freezing time, number of freeze-thaw cycles, and coal moisture content) and freeze-thaw damage variable *D*. The relationship between freeze-thaw damage variable *D* and freezing time *T* is exponential as is the relationship between *D* and the number of freeze-thaw cycles *C*. The relationship between *D* and the coal’s moisture content, *w*, is linear. The equations for these relationships are shown below.

2 $${D}_{T}={\rm{0.126}}-{\rm{0}}{{\rm{.11e}}}^{-{\rm{0.03}}T}\quad \quad ({{\rm{R}}}^{2}={\rm{0}}\mathrm{.903})$$

3 $${D}_{C}=-{\rm{0.013}}+{\rm{0}}{{\rm{.017e}}}^{{\rm{0.1}}C}\quad \quad ({{\rm{R}}}^{2}={\rm{0}}\mathrm{.954})$$

4 $${D}_{w}=0.38-0.025w\quad \quad ({{\rm{R}}}^{2}=0.867)$$

Figure 4(d) shows that freeze-thaw damage due to longer freezing times basically stops increasing when *D* reaches a value of about 0.12. This appears to be the limit for coal damage from freezing time alone. However, *D* continues to rise with an increase in the number of freeze-thaw cycles and the rate of damage suffered by the coal increases after 20 freeze-thaw cycles. The variable *D* shows a negative correlation with moisture contents and this suggests that the lower the moisture content, the more seriously the coal has been damaged in the elastic stage of deformation, and the result is consistent with the results from the acoustic emissions measurements. Because the maximum saturated moisture content for coal is with the 10–15% range, the influence of moisture contents on freeze-thaw damage is restricted by the fully saturation condition.

### Fractal characteristics of frozen-thawed coal

Fractal dimension is an important parameter for characterizing fracture distribution and connectivity of coal and rocks. Surface geometries of coal fractures can be quantitatively described by fractal dimension. The surfaces of fractures generated in the coal during freeze-thaw cycles were studied using fractal geometry. Fractal dimension can be calculated by several methods including correlation, similarity, capacity, information dimension, and box dimension^27–31^. Among these methods, box dimension based on grid coverage is most widely used for quantitatively analyzing fracture surfaces^30^.

Box dimension covers the surface of fractures by applying grids with a side length of *δ* so as to calculate the number, N(*δ*), of fractures in the grids. Different N(*δ*) can be obtained by changing the side length of the grids. The methods for grid partitioning on the fracture surfaces of frozen-thawed coal and fracture calculation are provided in Supplementary Fig. S2. Under a log-log coordinate system, regression analysis was conducted on the N(*δ*) of fractures and side length *δ* of grids using the least square method. The slope of the regression line represents the fractal dimension of the fracture surfaces^30^. The regression equation is shown as equation (5):

5 $$\mathrm{ln}\,N(\delta )=\,\mathrm{ln}\,A-{\rm{Dln}}\delta $$

where, A represents the number of original cracks.

Fracture density is an important parameter for evaluating the number and the distribution of fractures on coal surfaces. Fracture density *ρ* can be calculated by dividing the total fracture length ${\sum }_{{\rm{i}}=1}^{n}{L}_{i}$ of a section by the section area^30^. Thus

6 $$\rho =\sum _{i=1}^{{\rm{n}}}{L}_{i}/S$$

where, *L*_*i*_ represents the length of *i*th fracture, *n* is the total number of fractures, and *S* is the section area.

**Figure 5:**
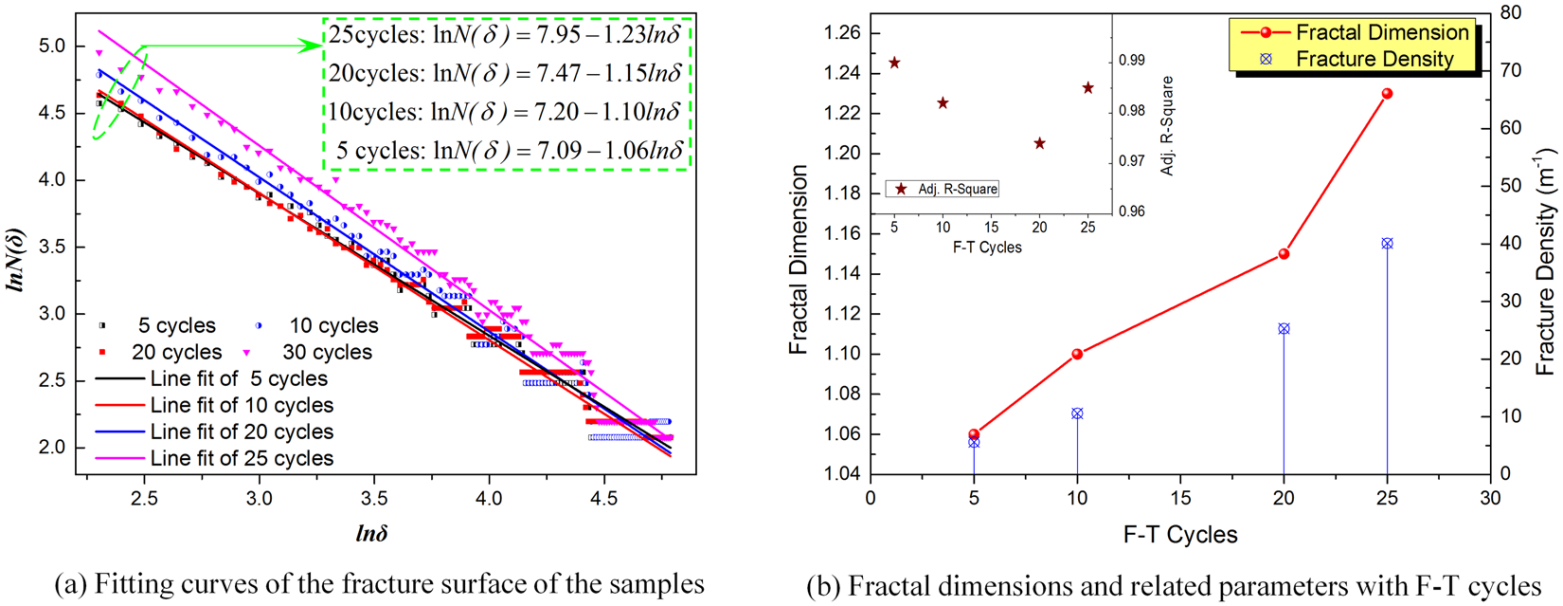
Fractal dimension of the coal freeze-thaw cycle fracture surfaces.

The fractal data for fractures from the freeze-thaw cycles were fitted to regression lines from equation (5) to obtain the fractal dimension of fracture surfaces. These regression lines are shown in Fig. 5(a). By carrying out quantitative analysis of the cracks on the coal surfaces, the fractal parameters for the freeze-thaw cycle fractures were determined. Fractal parameters for the surfaces of fractures induced by freeze-thaw cycling are listed in Supplementary Table S4.

It can be seen from Fig. 5 that the number of fractures on coal surfaces gradually increases as the number of freeze-thaw cycles increases. The fracture density grew from 1.06 m^−1^ for experiments with five freeze-thaw cycles to 40.1 m^−1^ for experiments with 25 cycles, and the fractal dimension increased from 1.06 to 1.23, with a constant increase trend. In addition, the regression coefficients were all larger than 0.97, which suggested that box dimension can reliably evaluate the fractal dimension for fractures in the frozen-thawed coal, as shown in Fig. 5(b). Because the fracture density and the fractal dimension rose with the increasing number of freeze-thaw cycles, it must be concluded that the number of freeze-thaw induced fractures in the coal were increased by the freeze-thaw cycles and the mechanical strengths of the coal was decreased. This provides favorable conditions for extracting CBM due to the increase of fracture permeability.

### Fracturing mechanism of LN_2_ freeze-thaw

**Figure 6:**
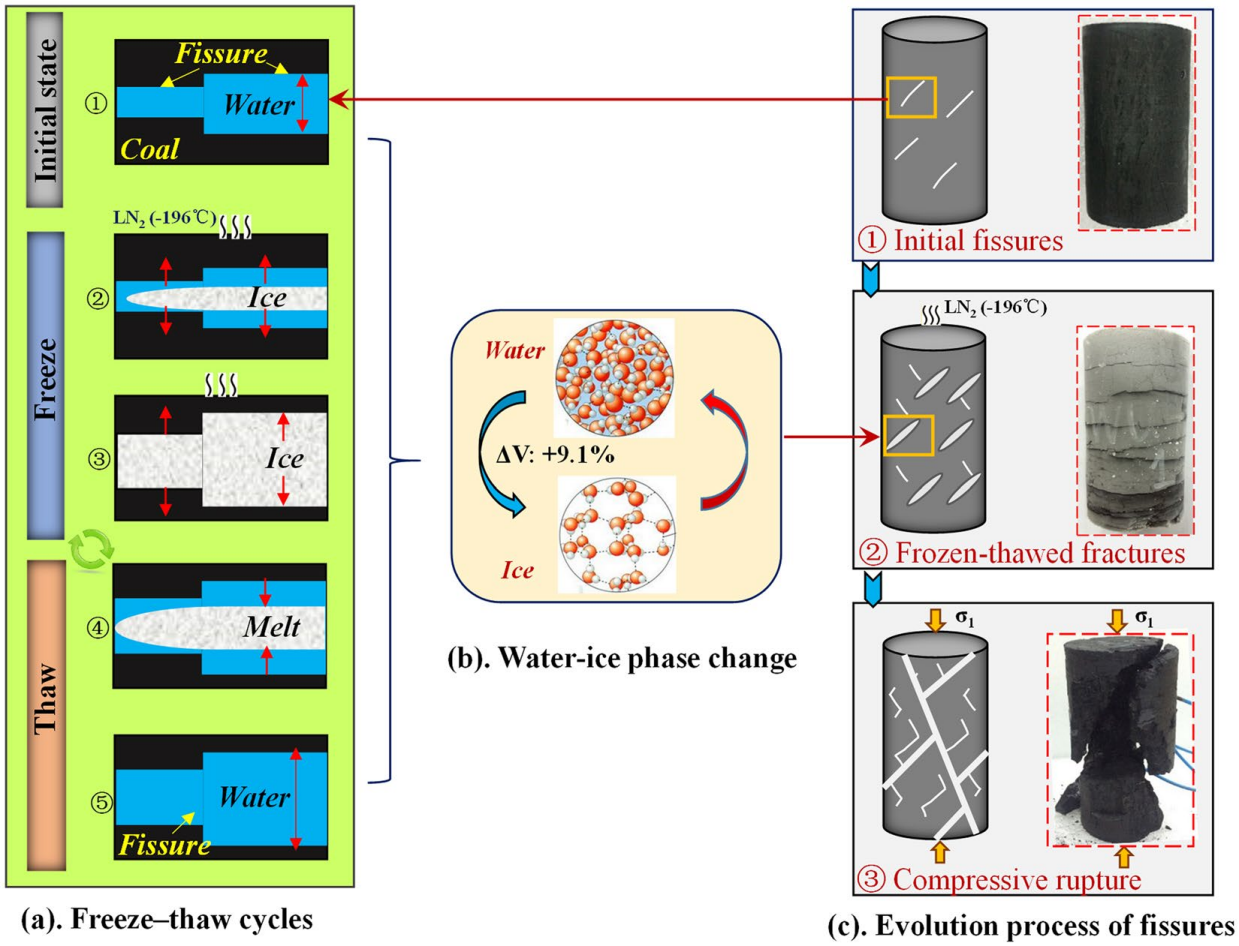
Modes of crack generation and extension in coal when the coal is treated with LN_2_.

The liquid-gas (N_2_) and water-ice (H_2_O) phase transitions after pumping LN_2_ into a coal seam causes three fracturing effects through expansion, frost heaving, and low-temperature fracturing. Because a few water-bearing initial fractures existed in the original coal, water in the fractures froze and expanded (Fig. 6(b)) after LN_2_ was poured on the coal. At the same time, the liquid nitrogen vaporized to gaseous nitrogen with nearly 296 times the volume of LN_2_. This meant that ice and the high-pressure nitrogen induced tension at the tips of fracture (Fig. 6(a)). Then the damage initiated by the nitrogen’s low-temperature caused the coal substrate to shrink non-uniformly, so tensile-shear stresses developed. The current stress loading from freeze-thaw cycles caused the formation of numerous freeze-thaw induced fractures (Fig. 6(a) and (c)). The mechanical properties of the coal declined due to the production and connection of freeze-thaw induced fractures. Under such conditions, a tiny external force can bring failure and deformation of the coal and form complex seepage networks for CBM, as shown in Fig. 6 (c) -③.

### Longitudinal wave velocities and permeability of coal after freeze-thawing

**Figure 7:**
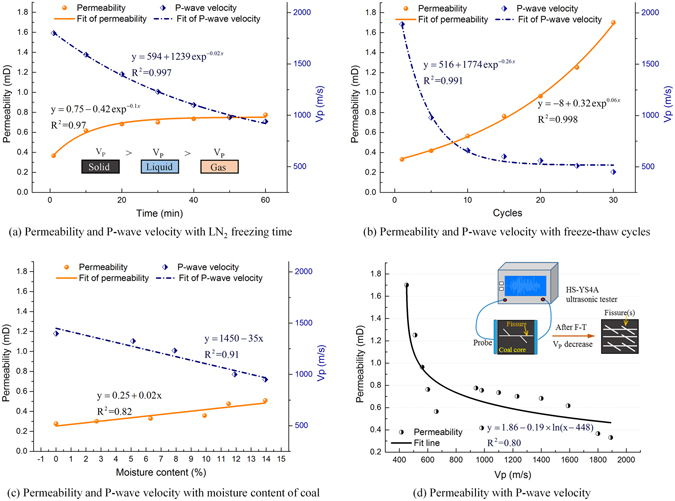
Graphs showing the correlation between longitudinal acoustic wave velocities and the permeability of coal subjected to different freeze-thaw treatments.

Ultrasonic testing is an important method for detecting the damage inside materials^32^. The propagation velocity of acoustic waves decreases successively in solids, liquids, and gases^33^, as shown in Fig. 7(a). For coal samples, the longitudinal velocity of ultrasonic waves is mainly influenced by the numbers of internal cracks. For superficially identical coal samples, lower ultrasonic wave velocities in one sample suggest that there are more fractures in that coal sample than the sample with higher wave velocities^34^. Therefore, acoustic wave velocities can be used to quantitatively describe the development of fractures in coal and thereby analyze the damage caused by freeze-thawing coal. Wyllie *et al*.^35^ established a time averaged model showing the relationship between wave velocity *v*_*p*_ and porosity *φ* in porous media. Wyllie *et al*.’s equation is^35^:

7 $${v}_{p}={(\frac{(1-\phi )}{{v}_{m}}+\frac{\phi }{{v}_{f}})}^{-1}$$

where, *v*_*p*_, *v*_*m*_ and *v*_*f*_ represent the equivalent wave velocity of coal, the wave velocity of solid matrix, and the wave velocity of fluid in pores, respectively.

According to equation (7), the expression for porosity can be inferred, as shown in equation (8).

8 $$\phi =\frac{({v}_{m}-{v}_{p}){v}_{f}}{({v}_{m}-{v}_{f}){v}_{p}}$$

In equation (8), *v*_*m*_ and *v*_*f*_ are fixed values in the same coal mass and fluid. The wave velocity *v*_*p*_ has a negative correlation with the porosity *φ*. In other words, the smaller the acoustic wave velocity in the coal, the greater the porosity of coal.

Permeability, the capability of a porous medium to transmit fluids, is also an important factor for evaluating the production capacity of CBM reservoirs. Permeability of coal shows an absolute positive correlation with the distribution and connectivity of fractures^36^. Figure 7 illustrates this correlation between ultrasonic wave velocity and permeability of frozen-thawed coal samples subjected to different freeze-thaw treatments.

As show in in Fig. 7, when the freezing time, *T*, number of freeze-thaw cycles, *C*, and moisture content in the coal, *w*, increase, the permeability improves and the longitudinal wave velocities in the frozen-thawed coal decreases. The cracks inside the coal gradually develop as *T*, *C* and *w*, which result in the wave velocity *V*_*P*_ decreases with these variables. To be specific, the wave velocity *V*_*P*_ decreased exponentially for longer freezing times and greater numbers of freeze-thaw cycles but velocities decline linearly with the coal’s water content. These relationships can be empirically regressed into equations (9)–(11). The trend for wave velocities to decline was more significant for the number of freeze-thaw cycles than for single freezing treatments.

9 $${V}_{p}=594+1239{\exp }^{-0.02T}\quad \quad {(R}^{2}=0.99)$$

10 $${V}_{p}=516+1774{\exp }^{-0.26C}\quad \quad {(R}^{2}=0.99)$$

11 $${V}_{p}=1450-35w\quad \quad {(R}^{2}=0.91)$$

Permeability *K* increased exponentially with *T* and *C* and linearly with *w* as expressed by equations (12)–(14). The permeability of the frozen-thawed coal remained below 0.8 md for a single freezing treatment. Compared to a single freezing time, the number of freeze-thaw cycles exerted a more significant effect on permeability. The permeability of coal reached a maximum of 1.7 md after 30 freeze-thaw cycles, an increase of 580% compared with the permeability of untreated coal. Because of the coal’s saturation limit, moisture content in the coal exerted a less significant influence on its permeability.

12 $$K=0.75-0.42{\exp }^{-0.1T}\quad \quad {({\rm{R}}}^{2}=0.97)$$

13 $$K=-8+0.32{\exp }^{0.06C}\quad \quad {({\rm{R}}}^{2}=0.99)$$

14 $$K=0.25+0.02w\quad \quad {({\rm{R}}}^{2}=0.82)$$

In general, coal permeability is positively correlated with porosity^37, 38^. As expressed in equation (8), porosity is negatively correlated with longitudinal wave velocity, so permeability is also negatively correlated with longitudinal wave velocity. This conclusion is consistent with the regression curve shown in Fig. 7(d). The permeability of frozen-thawed coal decreased logarithmic for longitudinal wave velocity as expressed by equations (15).

15 $$K=1.86-0.19\times \,\mathrm{ln}({V}_{p}-448)\quad \quad {({\rm{R}}}^{2}=0.80)$$

The strength of coal can be reduced by freeze-thaw damage within a certain range depending on the freezing time and the moisture content. However, the freeze-thaw cycles contributed to progressive coal damage. This is because many more freeze-thaw induced fractures are formed in the coal by the repeated freeze-thaw cycles than those by a single freezing event. The cycles result in increased porosity and permeability of the frozen-thawed coal (which decrease longitudinal wave velocity).

## Conclusions

Modifying samples using different liquid nitrogen (LN_2_) freeze-thaw treatments results in dissimilar damage to the coal samples. When the coal is frozen, the absolute values of axial and hoop strains in the frozen-thawed coal decrease as freezing time increase, the number of freeze-thaw cycles, and moisture contents in the coal increase. The uniaxial compressive strengths and elastic moduli of the coal samples are negatively correlated with freezing time and the number of freeze-thaw cycles and positively correlated with the coal’s moisture content. However, the coal’s Poisson’s ratio shows contrary results. The uniaxial test results are consistent with the acoustic emission data. The results allowed equations quantifying the changes caused by the three different freeze-thaw variables to the mechanical properties of the coal to be written.

With increasing freezing time, the value of the freeze-thaw damage variable *D* increases to about 0.12 but does not rise above that value. Variable *D* is negatively correlated with the coal’s moisture contents which shows that the lower the moisture content is, the larger the amount of damage done in the elastic stage of deformation. However, the upper limit of freeze-thaw damage related to moisture content that the coal can sustain is controlled by the coal’s water saturation limit.

Using the box counting method on fractures in the coal shows that fractal dimension increases from 1.06 for samples frozen-thawed through five freeze-thaw cycles to 1.23 for samples subject to 25 cycles. The direction in which the fractal dimension changed is always positive. The ultrasonic wave velocities exhibit a negative correlation with coal permeability and show that the freeze-thaw cycles have a significant effect on increasing the frozen-thawed coal’s permeability. Freeze-thaw fracturing with LN_2_ shows numerous advantages for modifying coal reservoirs and therefore is expected to become one of the important technologies for developing CBM resources efficiently.

## Methods

The coal samples used for these experiments were lignite collected from the Shengli coalfield in Inner Mongolia, China. The maceral contents and proximate analyses for the coal samples are listed in Supplementary Table S1. The coal samples were cores all drilled from the same large block of coal and all the cores were drilled in standard size 50 mm in diameter and 100 mm in length. The physical properties of the samples were tested using wave velocity and the samples with similar properties were selected for the experiments. Sample parameters and numbers are shown in Supplementary Table S2. The physical and mechanical properties of the samples used in the experiments are shown in Supplementary Table S3.

There are three main factors that control how freeze-thawing modifies and fractures coal. They are freezing time, number of freeze-thaw cycles, and the moisture content of coal. After being numbered, the coal samples were treated in a vacuum saturation device until they were fully saturated with water. Coal samples with different moisture contents were then produced by drying them for different lengths of time in a vacuum drying oven. Then samples with different moisture contents, including some completely saturated samples, were frozen separately for 1, 5, 10, 20, 30, 40, 50, and 60 min. Another set of samples was used for freeze-thaw experiments. The freeze-thaw experiments were conducted for 1, 5, 10, 15, 20, 25 and 30 cycles, each cycle involving 5 min of freezing and 5 min of thawing at room temperature. A third set of coal samples with different water contents were frozen for 90 min. These samples had moisture contents of 0%, 5.13%, 7.9%, 11.9%, and 13.9%. Following these freezing procedures, uniaxial compression tests were conducted on the samples. These compression tests included strain monitoring and the collection of acoustic emission signals during the compression tests and were conducted with the equipment shown in Supplementary Fig. S1.

## Electronic supplementary material

Factors controlling the mechanical properties degradation and permeability of coal subjected to liquid nitrogen freeze-thaw
