# Supplementary material for: Factors controlling the mechanical properties degradation and permeability of coal subjected to liquid nitrogen freeze-thaw
Source: Sci Rep. 2017 Jun 16;7:3675. doi: 10.1038/s41598-017-04019-7 (PMC5473936; doi:10.1038/s41598-017-04019-7)
Supplement: Supplementary file 1 — Factors controlling the mechanical properties degradation and permeability of coal subjected to liquid nitrogen freeze-thaw [file 41598_2017_4019_MOESM1_ESM.pdf]

Factors controlling the mechanical properties degradation and permeability of coal subjected to liquid nitrogen freeze-thaw

Lei Qin [1,2], Cheng Zhai [1,2*], Shimin Liu [3] & Jizhao Xu [1,2]

[1] Key Laboratory of Coal Methane and Fire Control，Ministry of Education，China University of Mining and Technology, Xuzhou, Jiangsu, 221116, China.

[2] School of Safety Engineering, China University of Mining and Technology, Xuzhou, Jiangsu, 221116, China.

[3] Department of Energy and Mineral Engineering, G[3] Center and Energy Institute, Pennsylvania State University, University Park, Pennsylvania 16802, United States.

* *corresponding author:* C.Z (email: greatzc@cumt.edu.cn)

Supplementary Figures

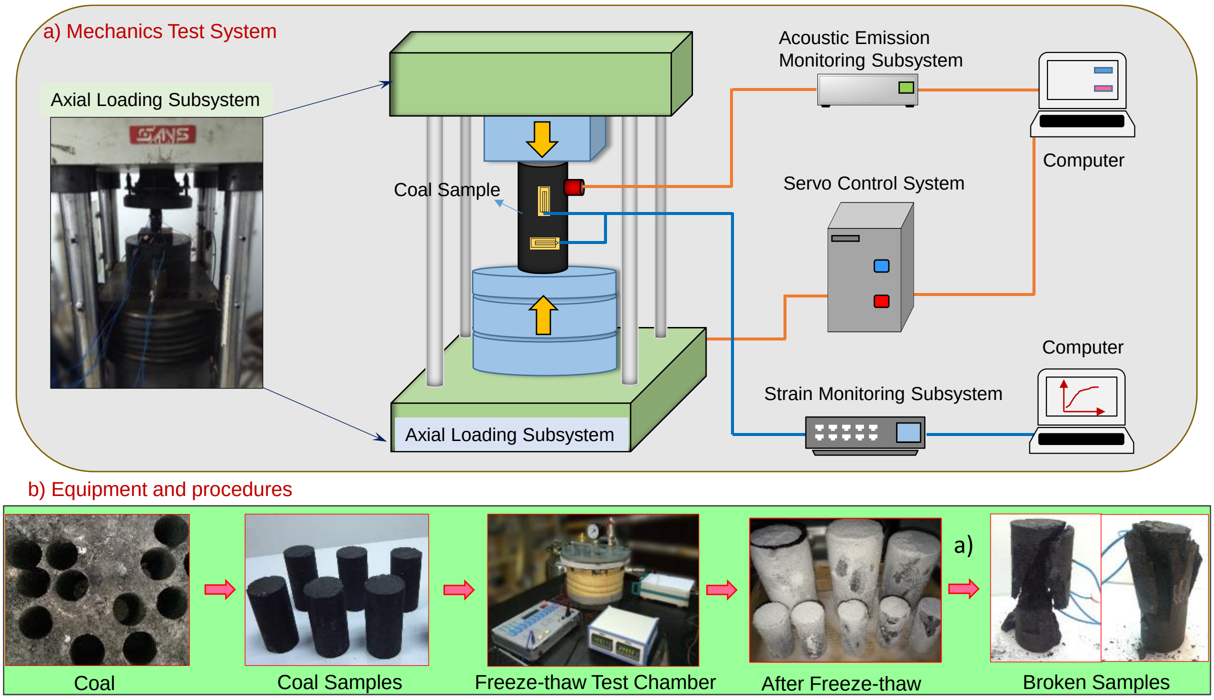

Supplementary Fig. S1. Diagrammatic illustration showing the experimental equipment and procedures.

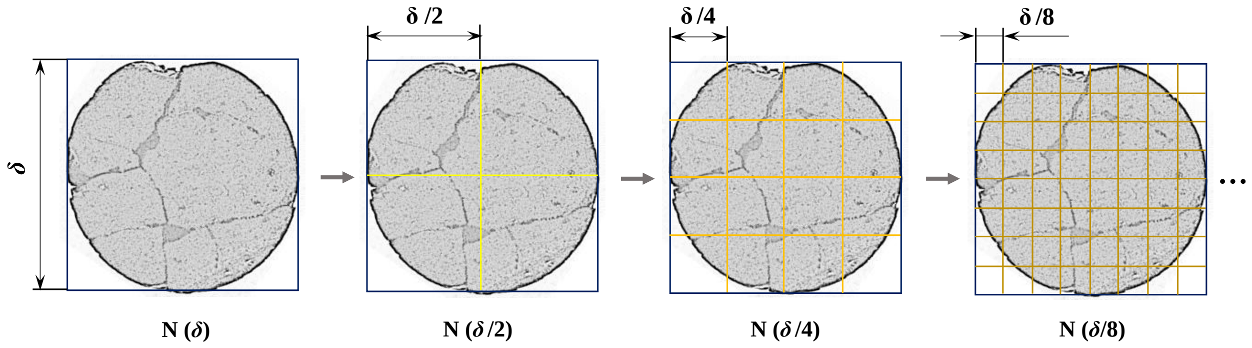

Supplementary Fig. S2. Methods for calculating fractal dimension on the fracture surfaces of the frozen-thawed coal samples.

Supplementary Tables

Supplementary Table S1. Macerals contents and proximate analyses of the coal samples.

Coal specimens	Maceral composition (vol %)				$R_{o,max}$ (%)	Proximate (wt %)			
	V	I	E	M		M_{ad}	A_{ad}	V_{daf}	FC_{ad}
Lignite	80.5	14.5	3.7	1.3	0.331	10.67	14.53	43.5	68.7

Notes: V, vitrinite; I, inertinite; E, exinite; M, minerals; M_{ad}, moisture, air-drying basis; A_{ad}, ash yield, air-drying basis; V_{daf}, volatile matter, dry ash-free basis; FC_{ad}, fixed carbon content, air-drying basis.

Supplementary Table S2. Sample Parameters and Numbers.

Sample	Height (mm)	Moisture contents (wt %)	Sample	Height (mm)	Moisture contents (wt %)
T−1	101.6	12.5	C−15	100	14.6
T−5	100	13	C−20	100.7	12.8
T−10	100.5	13.7	C−25	100	13.2
T−20	100	14.1	C−30	100.6	9.8
T−30	100.5	9.8	M−0	102	0
T−40	102	13.0	M-1	101.5	5.13
T−50	101	12.3	M-2	101	7.90
T−60	101	12.0	M-3	101	11.9
C−5	100.1	13.6	M-4	101.5	13.94
C−10	101	11.5	–	–	–

Notes: T−1 represents a coal sample that was frozen in LN_2 for 1 min then thawed at room temperature for 1 min (one freeze−thaw cycle); C−10 represents a coal sample that was frozen in LN_2 for 5 min then thawed at room temperature for 5 min (10 freeze−thaw cycles); M-1 represents a coal sample with a certain moisture contents; wt., weight percentage.

Supplementary Table S3. Physical and mechanical properties of samples used in the experiments.

Coal specimen	Density (kg/m^3)	Compressive strength (MPa)	Elastic modulus (GPa)	Poisson 's ratio /μ	Tensile strength (MPa)	Cohesion (MPa)	Internal friction angle (°)
Lignite	1161	11.2	0.51	0.28	0.15	0.18	20

Supplementary Table S4. Fractal parameters for the surfaces of fractures induced by freeze-thaw cycling in coal.

Cores no.	Num. F	Num. BF	FD(m^{-1})	FDFS	Adj. R-Square
5	1	2	5.6	1.06	0.990
10	2	3	10.7	1.10	0.982
20	4	4	25.3	1.15	0.974
25	8	7	40.1	1.23	0.985

Notes: Num. F-the number of main fractures; Num. BF-the number of breakage fragments; FD-fracture density; FDFS-the fractal dimension of fracture surface.

Supplementary Equipment

A SANS-YAW computer control pressure testing machine (MTS Systems Corporation, Shanghai, China) was used to complete the compression tests. This testing system has a maximum load of 3000kN, a load resolution of 1/300000 and a load relative error of ± 1%. The press loading speed is 0.05mm/min.

AEwin 8-channel acoustic emission data acquisition system (Physical Acoustics Corporation, Princeton Jct., New Jersey, USA) was used to collect the acoustic emission signals during compression tests. Acoustic emission sensor is NANO-30. The frequency range of the sensor is 100Hz~1MHz, and peak frequency is 230 kHz. Threshold of the collected data is 40db.

A vacuum drying oven (DZF-6020) and a vacuum water-saturation device were also used. An acoustic parameter tester (HS-YS4A) was used to measure P-wave velocities of ultrasonic waves in the coal.

Supplementary Experiment Procedures

After the coal samples were numbered, the samples were saturated with water in a saturation device. The weight and porosity of the samples were then measured.

The water-saturated samples were frozen separately for 1, 5, 10, 20, 30, 40, 50, and 60 min in the test chamber using LN_2 (see Supplementary Fig. S1 online). Freeze-thaw treatments were conducted on each frozen sample for 1, 5, 10, 15, 20, 25, and 30 cycles. A freeze-thaw cycle consists of 5 min of freezing followed by 5 min of thawing at room temperature.

Coal samples with different moisture contents were then produced by drying them for different lengths of time in a vacuum drying oven. A different set of coal samples with moisture contents of 0%, 5.13%, 7.9%, 11.9% and 13.94%, were also tested. All these coal samples were frozen for 90 min. Following these freezing procedures, uniaxial compression tests were conducted on the samples. These compression tests included strain monitoring and the collection of acoustic emission signals during the compression tests and were conducted with the equipment shown in Supplementary Fig. S1.
